# The Complete Mitochondrial Genome of *Ctenoptilum vasava* (Lepidoptera: Hesperiidae: Pyrginae) and Its Phylogenetic Implication

**DOI:** 10.1155/2012/328049

**Published:** 2012-04-05

**Authors:** Jiasheng Hao, Qianqian Sun, Huabin Zhao, Xiaoyan Sun, Yonghua Gai, Qun Yang

**Affiliations:** ^1^College of Life Sciences, Anhui Normal University, Wuhu 241000, China; ^2^SKLPS, LPS, Institute of Geology and Palaeontology, Chinese Academy of Sciences, Nanjing 210008, China; ^3^Department of Ecology and Evolutionary Biology, University of Michigan, Ann Arbor, MI 48109, USA

## Abstract

We here report the first complete mitochondrial (mt) genome of a skipper, *Ctenoptilum vasava* Moore, 1865 (Lepidoptera: Hesperiidae: Pyrginae). The mt genome of the skipper is a circular molecule of 15,468 bp, containing 2 ribosomal RNA genes, 24 putative transfer RNA (tRNA), genes including an extra copy of trnS (AGN) and a tRNA-like insertion trnL (UUR), 13 protein-coding genes and an AT-rich region. All protein-coding genes (PCGs) are initiated by ATN codons and terminated by the typical stop codon TAA or TAG, except for COII which ends with a single T. The intergenic spacer sequence between trnS (AGN) and ND1 genes also contains the ATACTAA motif. The AT-rich region of 429 bp is comprised of nonrepetitive sequences, including the motif ATAGA followed by an 19 bp poly-T stretch, a microsatellite-like (AT)_3_ (TA)_9_ element next to the ATTTA motif, an 11 bp poly-A adjacent to tRNAs. Phylogenetic analyses (ML and BI methods) showed that Papilionoidea is not a natural group, and Hesperioidea is placed within the Papilionoidea as a sister to ((Pieridae + Lycaenidae) + Nymphalidae) while Papilionoidae is paraphyletic to Hesperioidea. This result is remarkably different from the traditional view where Papilionoidea and Hesperioidea are considered as two distinct superfamilies.

## 1. Introduction

 The taxonomic status and the phylogenetic position of skippers (Hesperiidae) within Lepidoptera remain a controversial issue [1–3]. Due to the distinct differences between the skippers and the typical butterflies/moths in terms of morphological and behavioral characteristics, such as the short stout bodies, hooked antennae, and rapid skipping flight, the skippers were previously proposed to represent a separate group that is distinct from butterflies/moths in lepidopterans. More specifically, the skippers are assigned to the family Hesperiidae in a monotypic superfamily Hesperioidea, a sister lineage to the typical rhopaloceran butterflies, which mostly belong to superfamily Papilionoidea (true butterflies) [2, 4, 5]. In addition, the three superfamilies Hesperioidea, Papilionoidea, and Hedyloidea share numerous morphological characteristics, particularly in their egg, larval, and pupal stages, and thus were considered to be a large natural group [2].

The Lepidoptera is one of the largest groups of insects, accounting for more than 160,000 species. Despite of the huge taxonomic diversity, the current information on the lepidopteran mt genomes is very limited. Only 40 lepidopteran mt genomes were sequenced, including 10 butterfly species such as *Coreana raphaelis* [6], *Artogeia melete* [7], *Parnassius bremeri* [8], *Acraea issoria* [9], *Pieris rapae* [10], *Eumenis autonoe* [11], *Calinaga davidis* [12], and nearly thirty moth species such as *Bombyx mori, Bombyx mandarina* [13], *Adoxophyes honmai* [14], *Antheraea pernyi* [15], *Ochrogaster lunifer* [16], *Manduca sexta* [17], *Phthonandria atrilineata* [18], *Eriogyna pyretorum* [19], *Antheraea yamamai* [20], and *Caligula boisduvalii* [21]. The sampling is restricted to only six superfamiles (Papilionoidea, Totricoidea, Bombycoidea, Geometroidea, Noctuoidea, and Pyraloidea) to date, a complete mt genome sequence of a skipper from the family Hesperiidae is lacking, despite of a huge diversity of the skippers (>3500 species). The lack of mt genome data from skippers dampens phylogenetic and population genetic studies in skippers and the related species.

The Tawny Angle, *Ctenoptilum vasava*, is a typical skipper commonly found in southern East Asia, such as China, India, Burma, Thailand, and Vietnam. In this study, we sequenced the complete mitochondrial genome of the skipper, representing the first mt genome sequence from the family Hesperiidae (superfamily Hesperioidea). We next compared this sequence with other lepidopteran mt genomes sequences and examined the phylogenetic relationships within lepidopterans and reevaluated the phylogenetic position of skippers. We show that the skipper shares the general organization and structure of the mt genome with other species from the order Lepidoptera. By examining currently available mt genomes in lepidopterans, we find the Hesperioidea is placed within the Papilionoidea, which may be a paraphyletic group. 

## 2. Materials and Methods

### 2.1. Sample Collection

Adult individuals of *Ctenoptilum vasava* (Lepidoptera: Hesperiidae: Pyrginae: *Ctenoptilum*) were captured from National Natural Conservation Areas of Jiu Lianshan Mountain, Jiangxi Province, China, in August, 2009. After a brief examination for species identification, the fresh tissues were preserved in 100% ethanol immediately for DNA fixation and stored at −20°C until further use for genomic DNA isolation.

### 2.2. DNA Extraction and PCR Amplification

The whole genomic DNA of *C. vasava* was isolated from the thoracic muscle of an adult individual using the proteinase K-SiO_2_ method as described by Hao et al. [32]. Partial sequences of COI, COIII, Cytb, ND4, 16S rRNA, and 12S rRNA genes were amplified using insect universal primers [33]. Polymerase chain reactions (PCRs) were performed under the following condition: 5 minutes of initial denaturation at 95°C, 35 cycles of denaturation at 95°C for 50 seconds, annealing at 45–55°C (depending on primer pairs) for 50 seconds, extension at 72°C for 1 minute, and a final extension at 72°C for 10 minutes. Based on the sequences from the newly acquired gene fragments, the long PCR primers were designed (Table 1) according to the conserved regions by the program Primer premier 5.0 [34], and the entire mt genome of the skipper was in turn amplified in five long fragments (12S-COI, COI-COIII, COIII-ND4, ND4-Cytb, Cytb-12S) by using Takara LA TaqTM (Takara). The long PCR condition is as follow: an initial denaturation at 95°C for 5 min, 15 cycles of denaturation at 95°C for 50 seconds, annealing at 50–55°C (depending on primer pairs) for 50 seconds, extension at 68°C for 150 seconds, additional 15 cycles of denaturation at 95°C for 50 seconds, annealing at 50–55°C for 50 seconds, extension at 68°C for 150 seconds, and a final extension at 68°C for 10 minutes. PCR products were examined by electrophoresis on a 1% agarose gel and purified using a DNA gel extraction kit (Takara). All PCR fragments were directly sequenced in both strands after purification with QIA quick PCR Purification Kit (Qiagen). Long PCR fragments were sequenced using the primer walking strategy (walking primer information will be provided upon request).

### 2.3. Sequence Analysis

The raw sequence files were proof-read and assembled in BioEdit version 7.0 [35]. The concatenated amino acid sequences of the 13 PCGs were obtained and analysed by the ClustalX [36] and the MEGA 3.0 [37] softwares. The structures of the 23 tRNAs and a tRNA-like gene of the skipper were identified by the software tRNAscan-SE version 1.21 [38]. The putative tRNAs, which were not found by tRNAscan-SE, were identified by sequence comparisons between the skipper and other lepidopteran tRNAs. Nucleotide composition was calculated using MEGA 3.0 software [37], and the tandem repeats in the AT-rich region were predicted by the Tandem Repeats Finder available online (http://tandem.bu.edu/trf/trf.html) [39]. The mt genome sequence has been submitted to GenBank database under the accession number JF713818.

### 2.4. Phylogenetic Analysis

The multiple alignment of the 13 PCG concatenated nucleotide sequences of the 32 lepidopteran mt genome sequences (one is from this study, and 31 were extracted from GenBank, see Table 2) was conducted using Clustal X1.8 [36] and was checked by eye. The phylogenetic trees were reconstructed with the maximum likelihood (ML) and bayesian inference (BI) methods using a hymenopteran species Apis cerana (GenBank accession number NC_014295) as the outgroup. In both phylogenetic analyses, the third position of all the codons was excluded. The ML analyses were conducted in PAUP (version 4.0b8) [40] with searching method of TBR branch swapping (10 random addition sequences), the general time reversible model with invariant sites and among-site variation (GTR+I+Γ) was selected as the best fit model using Modeltest (version 3.06) [41] under the AIC criteria, and the bootstrap values of the ML tree were evaluated via the bootstrap test with 1000 iterations. The Bayesian analysis was performed using MrBayes 3.1.2 [42] with the partitioned strategy, and the best fit substituion model was selected as in the ML analysis. MrBayes 3.1.1 simultaneously initiates 2 Markov Chain Monte Carlo (MCMC) that runs to provide additional confirmation for the convergence of posterior probability distribution. Four simultaneous chains were run, 3 hot and 1 cold. Analyses were run for one million generations until the average standard deviation of split frequencies to be less than 0.01, which means convergence was reached. Chains were sampled every 1,000 generations. Starting trees were random.

## 3. Results and Discussion

### 3.1. Genome Organization, Gene Arrangement, and Base Composition

**Figure 1:**
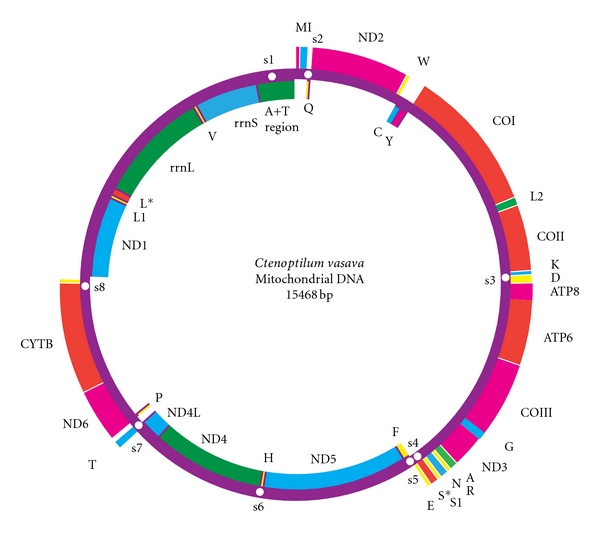
Map of the circular mitochondrial genome of *Ctenoptilum vasava*. Genes encoded in the H-strand (clockwise orientation) are colored in red or pink. Genes encoded in the L-strand (anticlockwise orientation) are colored in blue or green. The abbreviations for the genes are as follows: *COI-III* stands for cytochrome oxidase subunits, *Cytb* for cytochrome b, and *ND1-6* for NADH dehydrogenase components. *tRNAs* are denoted as one-letter symbol according to the IUPAC-IUB single-letter amino acid codes.

The organization of the skipper mt genome was shown in Figure 1. The complete mt genome is 15, 468 bp in length, containing 13 protein-coding genes (ND1-6, ND4L, COI-III, Cytb, ATP6, ATP8), 2 ribosomal RNAs (12S and 16S), 24 putative tRNAs, and an AT-rich region, same characteristics as with those of other butterfly species available (Tables 3 and 4). The size of so far sequenced mt genomes in lepidopteran insects ranges from 15,140 bp in *Artogeia melete* [7] to 15,928 bp in *Bombyx mandarina* [13]; our newly sequenced mt genome of the skipper is within the above size range. Mitochondrial genes of the skipper are arranged in the same order and orientation as those of other lepidopterans, except for the presence of an extra copy of trnS (AGN) and a tRNA-like insertion trnL(UUR). Similar to many insect mt genomes, the heavy strand (H-strand) encodes more genes (9 PCGs and 14 tRNAs), whereas the light strand (L-strand) encodes less (4 PCGs, 8 tRNAs, and 2 rRNA genes). 

The composition of A, T, G, C nucleotides in the skipper mt genome L-strand is 39.09%, 41.45%, 7.73%, and 11.72%, respectively, indicating a remarkably high AT content (80.55%) and a low GC content (19.45%). This result is consistent with the AT bias generally observed in other lepidopteran mt genomes, given that the composition of AT is ranging from 77.9% in *Ochrogaster lunifer* [16] to 82.7% in *Coreana raphaelis* [6]. The AT and GC skew values in H-strand of the skipper mt genome are −0.0293 and −0.216, respectively, indicating that T and C are favored over A and G, respectively.

### 3.2. Protein-Coding Genes (PCG)

The 13 PCGs of the skipper mt genome include 7 NADH dehydrogenase subunits, 3 cytochrome c oxidase subunits, 2 ATPase subunits, and one cytochrome b gene. The 13 PCGs are 11,172 bp in length which accounts for 72.23% of the whole mitochondrial genome, encoding 3,724 amino acid residues. All the protein-coding genes except for the COI start with a canonical start codon ATN. More specifically, 7 PCGs (COII, ATP6, COIII, ND4, ND4L, Cytb, and ND1) start with ATG, 2PCGs (ND2, ND3) with ATT, additional 3 PCGs (ATP8, ND5, ND6) with ATA. For the stop codon, 3 PCGs (the ND2, ND3, and ND5) terminate with ATG, 2 PCGs (COI and COII) with a single T, the remaining 8 PCGs with the typical stop codon TAA.

The start codons for COI gene of the lepidopteran insects are not uniform. All lepidopteran species examined to date use Arginine (R), which correspond to the codon CGA, as the initial amino acid for COI [43]. In this study, the CGA is not conserved across all lepidopteran mt genomes, but it has been suggested to serve as the COI start codon [8]. However, in other insect groups, some other canonical codons, such as the TTA [44], TCG [45], TTG [46], ACG [47], were reported as the COI start codons. Additionally, some tetranucleotides, such as the ATAA, ATCA, and ATTA [48], as well as the hexanucleotides, such as the ATTTAA [49–51], TATCTA [52], TTTTAG [13], and TATTAG [29], were also proposed as the start codons for this gene.

The 13 protein-coding genes all possess complete stop codons except for the COI and COII in this study. Most stop codons for COII are single T in most sequenced lepidopteran mt genomes. The single T stop codon is usually located nearby the trnK, the structure of which is recognized by endonucleases processing the polycistronic pre-mRNA transcription, and produces functional stop codons by polyadenylation from its contiguous protein-coding genes [53–55]. Accordingly, partial stop codons observed in most lepidopteran species would be one strategy for the selection of stop codon; in other words, it would minimize the intergenic spacers and gene overlaps from an evolutionary economic perspective.

The amino acids composition and the codon usage of the skipper mt genome are shown in the Tables 5 to 6. The results showed that the codon usage of all the genes has a strong bias, and the RSCU (relative synonymous codon usage) of NNU and NNA codons are greater than 1, indicating that the third positions of the UA have a high frequency of codon usage. The codon usage bias in protein-coding genes and the third position of AT bias (92.1%) are positively correlated. In addition, our analysis also showed that UUU (Phe), UUA (Leu), AUU (Ile), AUA (Met), and AAU (Asn) are the most frequently used codons (45.45%). Together, all observations suggest the strong AT bias of the protein-coding genes in the skipper mt genome.

### 3.3. Ribosomal and Transfer RNA Genes

The srRNA (12S rRNA) and lrRNA (16S rRNA) genes in the skipper mt genome are 774 bp and 1343 bp in size, respectively. Similar to other lepidopteran rRNAs [8, 22], the skipper 12S rRNA is located between trnL (CUN) and trnV, whereas its 16S rRNA resides between trnV and the A+T-rich region. The A+T contents of the two rRNA genes are 86.43% and 84.14%, respectively, which is consistent with those observed in other lepidopterans as well.

**Figure 2:**
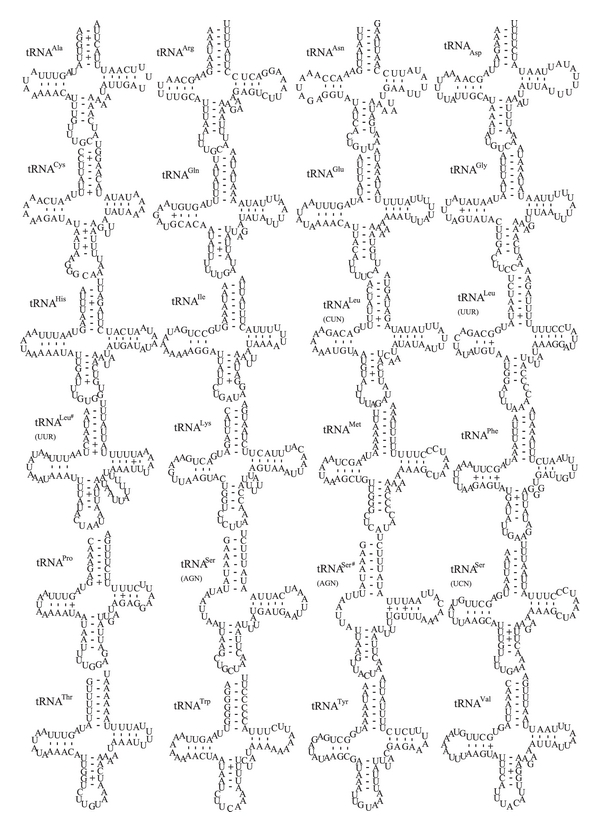
Predicted secondary clover-leaf structure for the tRNA genes of *Ctenoptilum vasava*. Dashes: Watson-Crick base pairing; centered dots (+): unmatched base pairing.

The skipper mt genome contains 23 transfer RNAs and a tRNA-like gene (trnL (UUR)), and these genes are interspersed throughout the whole genome and ranged in size from 61 to 79 bp. Twenty-three of them are shown to be folded into the expected clover-leaf secondary structure; however, the trnS (AGN) lacks the dihydrouridine (DHU) loop (Figure 2) as shown in the other insect mt genomes examined to date. Therefore, the incomplete trnS should be considered as one of common features in most, if not all, insect mt genomes. A total of 32 unmatched base pairs were detected in the skipper tRNAs, and 18 of them are GU pairs, which form a weak bond in the tRNAs, the remaining 14 are atypical pairs, including 11 UU pairs, two AC pairs, and one AA pairs (Figure 2). 

The extra trnS was detected between trnS (AGN) and trnE in the skipper mt genome (Figure 2), and the tRNA insertions were also observed in other lepidopterans, such as *C. raphaelis* and *A. issoria*. The high similarity between the two tandemly repeated copies of the trnS in primary and secondary structures may suggest a recent duplication event [6, 9]. Interestingly, a 79 bp insertion of a tRNA-like gene was detected between the trnL (CUN) and lrRNA genes, and this observation was not shown in any lepidopteran mt genomes to date, despite that tRNA translocation is a frequent event in the evolution of lepidopteran mt genomes [56].

### 3.4. Intergenic Spacers and Overlapping Sequences

The skipper mt genome contains a total of 607 bp intergenic spacers, which distribute in 11 regions, ranging from 2 to 61 bp in length. Most of the spacers are shorter than 10 bp, only 7 spacers are longer (Table 2). The longest intergenic spacer (61 bp) is located between the trnQ and ND2 genes, with a high AT content (98.4%). This spacer is usually considered as a constitutive synapomorphic feature of lepidopteran mt genomes because of the absence in nonlepidopteran insects [17]. The second longest one (ATACTAAAAATATATTA) is inserted between the trnS2 and ND1 and shared the ATACTAA motif observed in most insects, including lepidopterans [17], and possibly fundamental to site recognition by the transcription termination peptide (mtTERM protein) [57]. The third longest spacer (CAATTTCTTTT) is inserted between the trnC and trnY, which is the same as *O. lunifer*. The fourth longest spacer (AAATTATTAAATTT) is located between trnK and trnD, which is also found in other lepidopterans. The fifth longest one (TTTTCTTTTCTTT) is resided between the two trnS, which is almost identical to that in *C. raphaelis*.

The skipper mt genes are overlapped in a total of 51 bp at 12 locations, with the longest overlap of 7 bp, which is located between the ATP8 and ATP6. The overlapped nucleotides are shared in all the lepidopteran mt genomes so far examined and are probably helpful to form the structure of hairpin loop for posttranslation modifications [6, 43]. Other overlap regions include the 5 bp nucleotides between the trnW and trnC, 3 bp nucleotides between the trnY and COI, and the remaining 9 overlapping nucleotides are shorter than 3 bp (Table 4). Of the overlaps, the one between the trnF and ND5 is only 1 bp in size, substantially shorter than those of other lepidopteran species, such as the *A. pernyi* (15 bp),* C. boisduvalii* (17 bp), *E. pyretorum* (17 bp), *O. furnacalis* (15 bp), *O. nubilalis* (16 bp), *E. autonoe* (16 bp), and *A. honmai* (29 bp).

### 3.5. The AT-Rich Region

**Figure 3:**
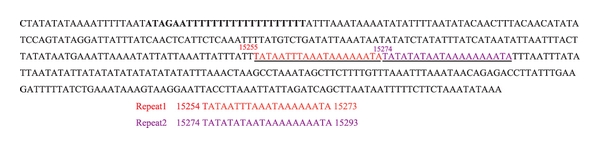
Repeated segments in A+T-rich region in the skipper mt genome. The consensus pattern (20 bp) repeat unit (TATAATTTAATAAAAAAAATA) is highlighted in red and purple. Numbers are shown as the positions of starting nucleotides.

The 429 bp AT-rich region with the AT content of 88.11% is located between the srRNA and trnM. This region includes the O_N_ (origin of minority or light strand replication) which has the motif ATAGA and is followed by 19 bp poly-T that is conserved across all lepidopterans. The AT-rich region also includes a few short microsatellite-like repeat regions such as poly-A, poly-T, and (AT)_3_ (TA)_9_ prior to the structural motif ATTTA or ATTA that is common to all the lepidopteran mt genomes. In addition, for the first time, we found two large repeated elements, the 20 bp repeat (TATAATTTAATAAAAAAAATA), in the AT-rich region in a lepidopteran mt genome (Figure 3), similar observation was also reported in satyrid *Eumenis autonoe*, despite that their functions still remain unknown [11].

## 4. Phylogenetic Analysis

A total of 32 lepidopteran species represent seven lepidopteran superfamilies (Tortricoidea, Pyraloidea, Papilionoidea, Hesperioidea, Bombycoidea, Geometroidea, and Noctuoidea). Of them, the superfamilies Papilionoidea and Hesperioidea are usually referred to as the macrolepidopterans together with the superfamilies Bombycoidea, Geometroidea, and Noctuoidea. Their phylogenetic relationships have long been a subject of controversy [58–61]. One of the most compelling hypotheses is that the Hesperioidea and Papilionoidea are closely related to each other [58, 60].

**Figure 4:**
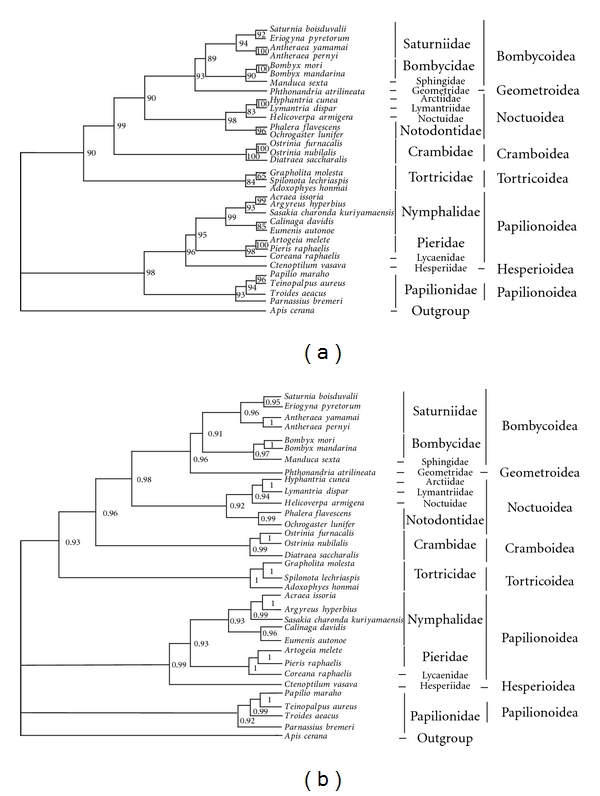
Phylogenetic trees of the lepidopterans based on 13 PCG nucleotide sequences. (a) ML tree. (b) BI tree. Numbers at each node indicate bootstrap percentages of 100 pseudoreplicates for ML analysis and posterior probability for BI analysis.

The ML and BI phylogenetic trees in this study based on 13 protein coding genes of the mitochondrial genomes revealed two major clusters: the first includes Bombycoidea, Geometroidea, Noctuoidea, Tortricoidea, and Cramboidea, and the second contains Papilionoidea and Hesperioidea with strong support (Figure 4(a)). Because the first cluster was also revealed by Kim et al. [11] and Mutanen et al. [62], we here focus on the second cluster, in which the superfamily Papilionoidea appears to be paraphyletic (Figure 4(a)) or polyphyletic (Figure 4(b)), and the superfamily Hesperioidea is placed within the Papilionoidea as a sister to ((Pieridae + Lycaenidae) + Nymphalidae). This result is consistent with those reported earlier based on combined data sets of a few nuclear and mitochondrial genes [62–64]. It is, however, remarkably different from the traditional view where Papilionoidea and Hesperioidea are considered as two distinct superfamilies. In conclusion, on the contrary to the common view that Papilionoidea and Hesperioidea are two separate groups in light of morphological and behavioral characteristics, our new mt genome data support that, while the majority of the Papilionoidea lineages is a sister group to Hesperioidea, the Papilionoidae is a separate clade among the two superfamilies. Our results call for more data from Hesperioidea to establish the accurate phylogeny of skippers (Hesperioidea) and true butterflies (Papilionoidea). 

## Figures and Tables

**Table 1:** Primer sequences for the long PCR amplification used in this study.

Primers	Upper primer sequence (5′-3′)	Lower primer sequence (5′-3′)
COI-COIII	GGAAATTGACTTGTGCCT	TTGTATGTTTACCTTGGA
COIII-ND4	AAAGGATTACGATGAGGT	GGTCTTGTTATTGGTGGA
ND4-Cytb	CGTCTATGTAAACGCTCA	ATAAGGGTTTTCTACTGGT
Cytb-12S	TTTTACATCAAACAGGA	ACTAGGATTAGATACCC
12S-COI	GAAACACTTTCCAGTACCT	CTAAACCAATTCAACATCC

**Table 2:** Lepidopteran mt genome sequences used in this study.

Species	Superfamily and family		GenBank acc. no.	References
*Hyphantria cunea*	Noctuoidea	Arctiidae	NC_014058	[22]
*Lymantria dispar*		Lymantriidae	NC_012893	[23]
*Helicoverpa armigera*		Noctuidae	NC_014668	[24]
*Ochrogaster lunifer*		Notodontidae	NC_011128	[16]
*Phalera flavescens*		Notodontidae	JF440342	(Sun et al., unpublished)
*Phthonandria atrilineata*	Geometroidea	Geometridae	NC_010522	[18]
*Spilonota lechriaspis*	Tortricoidea	Tortricidae	NC_014294	[25]
*Adoxophyes honmai*		Tortricidae	NC_008141	[14]
*Grapholita molesta*		Tortricidae	NC_014806	[26]
*Antheraea pernyi*	Bombycoidea	Saturniidae	NC_004622	[15]
*Antheraea yamamai*		Saturniidae	NC_012739	[20]
*Eriogyna pyretorum*		Saturniidae	NC_012727	[19]
*Caligula boisduvalii*		Saturniidae	NC_010613	[21]
*Chinese Bombyx mandarina*		Bombycidae	AY301620	[27]
*Bombyx mandarina*		Bombycidae	NC_003395	[13]
*Bombyx mori*		Bombycidae	NC_002355	[13]
*Manduca sexta*		Sphingidae	NC_010266	[17]
*Diatraea saccharalis*	Pyraloidea	Crambidae	NC_013274	[28]
*Ostrinia furnacalis*		Crambidae	NC_003368	[29]
*Ostrinia nubilalis*		Crambidae	NC_003367	[29]
*Eumenis autonoe*	Papilionoidea	Nymphalidae	NC_014587	[11]
*Acraea issoria*		Nymphalidae	GQ376195	[9]
*Sasakia charonda kuriyamaensis*		Nymphalidae	NC_014223	(Hakozaki et al., unpublished)
*Argyreus hyperbius*		Nymphalidae	JF439070	[30]
*Calinaga davidis*		Nymphalidae	HQ658143	[12]
*Papilio maraho*		Papilionidae	NC_014055	[31]
*Parnassius bremeri*		Papilionidae	NC_014053	[8]
*Teinopalpus aureus*		Papilionidae	NC_014398	(Qin et al., unpublished)
*Troides aeacus*		Papilionidae	EU625344	(Jiang et al., unpublished)
*Coreana raphaelis*		Lycaenidae	NC_007976	[6]
*Pieris raphaelis*		Pieridae	HM156697	[10]
***Ctenoptilum vasava***	Hesperoidea	Hesperiidae	JF713818	This study

**Table 3:** Comparison of the mt genome characteristics of all butterfly species available. All genomes share the features of 13 protein-coding genes (ND1-6, ND4L, COI-III, Cytb, ATP6, ATP8), 2 ribosomal RNAs (lsu rRNA (12S) and lsu rRNA (16S)), and an AT-rich region.

Taxon	Whole genomic DNA				lrRNA		srRNA		A+T-rich region		GenBank accession no.
	Size (bp)	A+T %	No. codons^a^	PCG^b^ A+T %	Size (bp)	A+T %	Size (bp)	A+T %	Size (bp)	A+T %	
*Adoxophyes honmai*	15,680	80.4	3,748	78.5	1,387	83.6	779	85.4	489	94.3	DQ073916
*Antheraea pernyi*	15,575	80.2	3,732	78.5	1,369	83.9	775	84.1	552	90.4	AY242996
*Antheraea yamamai*	15,338	80.2	3,714	79.3	1,380	83.5	776	85.9	334	90.4	EU726630
*Caligula boisduvalii*	15,360	80.6	3,734	79.1	1,391	84.8	774	84.1	330	91.5	EF622227
*Eriogyna pyretorum*	15,327	80.8	3,711	79.3	1,338	84.6	762	84.5	374	91.7	FJ685653
*Bombyx mori*	15,656	81.4	3,720	79.5	1,378	84.4	783	85.6	494	95.5	AB070264
*Manduca sexta*	15,516	81.8	3,718	80.2	1,391	84.7	777	86.8	324	95.1	EU286785
*Phthonandria atrilineata*	15,499	81.1	3,724	79.0	1,400	85.1	803	87.5	457	98.5	NC010522
*Parnassius bremeri*	15,389	81.3	3,734	80.2	1,344	83.8	773	85.1	504	93.6	FJ871125
*Artogeia melete*	15,140	79.8	3,715	78.4	1,319	83.4	777	86.9	351	88.0	NC010568
*Coreana raphaelis*	15,314	82.7	3,708	81.5	1,330	85.3	777	85.8	375	94.1	DQ102703
*Eumenis autonoe*	15,489	79.1	3,728	76.8	1,335	83.7	775	85.3	678	94.5	GQ868707
*Apatura ilia*	15,242	80.5	3,711	78.9	1,333	86.0	776	84.9	403	92.5	JF437925
*Calinaga davidis*	15,267	80.4	3,737	78.8	1,337	83.8	773	85.9	403	92.5	HQ658143
*Argyreus hyperbius*	15,156	80.8	4,164	79.7	1,330	84.5	778	85.2	349	95.4	JF439070
*Acraea issoria*	15,245	79.7	3,745	78.0	1,331	83.8	788	83.8	430	96.0	GQ376195
*Ochrogaster lunifer*	15,593	77.9	3,746	75.7	1,351	81.4	806	84.3	319	92.7	AM946601
*Lymantria dispar*	15,569	79.9	3,742	77.8	1,351	84.2	799	85.2	435	96.1	FJ617240
***Ctenoptilum vasava***	**15,468**	**80.5**	**3,698**	**78.9**	**1,343**	**84.1**	**774**	**86.4**	**429**	**88.1**	**JF713818**

**Table 4:** Summary of the skipper mt genome. IGNc stands for intergenic nucleotides, and the positive number indicates interval nucleotides (base pairs) between genes, while the negative number indicates the overlapped nucleotides (base pairs) between genes.

Gene	Direction	Nucleotide no.	Size	IGNc	Start codon	Stop codon
tRNA^Met^	F	1–68	68	0		
tRNA^lle^	F	69–133	65	0		
tRNA^Gln^	R	136–204	69	2		
ND2	F	266–1270	1005	61	ATT	TAG
tRNA^Trp^	F	1269–1335	67	−2		
tRNA^Cys^	R	1331–1395	65	−5		
tRNA^Tyr^	R	1407–1471	65	11		
COI	F	1469–2990	1522	−3	ATT	T-tRNA
tRNA^Leu^(UUR)	F	2991–3057	67	0		
COII	F	3058–3731	674	0	ATG	T-tRNA
tRNA^Lys^	F	3732–3801	70	0		
tRNA^Asp^	F	3816–3884	69	14		
ATP8	F	3885–4052	168	0	ATA	TAA
ATP6	F	4046–4723	678	−7	ATG	TAA
COIII	F	4723–5505	783	−1	ATG	TAA
tRNA^Gly^	F	5508–5574	67	2		
ND3	F	5575–5928	354	0	ATT	TAG
tRNA^Ala^	F	5927–5990	64	−2		
tRNA^Arg^	F	5996–6061	66	5		
tRNA^Asn^	F	6061–6125	65	−1		
tRNA^Ser^(AGN)	F	6133–6194	62	7		
tRNA^Ser^(AGN)	F	6208–6267	60	13		
tRNA^Glu^	F	6279–6344	66	11		
tRNA^Phe^	R	6343–6409	67	−2		
ND5	R	6409–8145	1737	−1	ATA	TAA
tRNA^His^	R	8161–8228	68	15		
ND4	R	8228–9568	1341	−1	ATG	TAA
ND4L	R	9574–9855	282	5	ATG	TAA
tRNA^Thr^	F	9869–9933	65	13		
tRNA^Pro^	R	9934–9998	65	0		
ND6	F	10001–10531	531	2	ATA	TAA
Cytb	F	10531–11679	1149	−1	ATG	TAA
tRNA^Ser^	F	11679–11745	67	−1		
ND1	R	11763–12704	942	17	ATG	TAA
tRNA^Leu^(CUN)	R	12706–12773	68	1		
tRNA^Leu^(UUR)	F	12774–12852	79	0		
16S	R	12853–14195	1343	0		
tRNA^Val^	R	14196–14265	70	0		
12S	R	14266–15039	774	0		
A+T-rich region		15040–15468	429	0		

**Table 5:** Amino acid composition of the mt genome of the skipper. Start codons and stop codons were excluded in total codon counts.

	Ala	Cys	Asp	Glu	Phe	Gly	His	Ile	Lys	Leu	Met	Asn	Pro	Gln	Arg	Ser	Thr	Val	Trp	Tyr	Total
ND2	2.10	1.20	0.30	1.50	17.72	3.30	0.60	13.81	5.11	11.11	8.41	8.41	1.50	1.20	0.90	12.01	3.00	1.20	2.40	4.20	333
COI	5.72	0.39	2.76	1.58	8.88	7.89	2.96	10.65	1.78	11.64	6.34	4.93	5.13	1.97	1.38	9.47	5.52	4.14	2.56	4.34	507
COII	3.59	0.9	3.59	4.48	7.17	2.69	2.24	13.9	2.24	12.11	4.48	9.87	4.48	4.04	3.14	6.73	4.48	3.59	2.69	3.59	223
ATP8	0.00	0.00	0.00	0.00	12.96	0.00	0.00	20.37	9.26	5.56	9.26	12.96	5.56	1.85	0.00	3.70	1.85	0.00	5.56	11.11	54
ATP6	2.23	0.00	0.45	1.79	9.82	4.46	2.23	12.95	0.89	17.41	4.91	7.14	4.91	2.23	1.34	11.16	7.14	4.02	2.23	2.68	224
COIII	5.02	0.77	1.54	2.70	11.20	6.56	5.02	12.74	1.54	10.42	2.70	4.25	3.86	2.32	1.54	6.18	7.72	3.47	5.02	5.41	259
ND1	2.88	1.28	2.24	3.21	10.26	5.13	0.00	9.62	3.53	18.59	5.13	4.49	1.92	1.28	1.60	11.86	3.21	4.49	1.92	7.37	312
Cytb	4.46	0.79	1.84	1.05	9.45	5.77	2.10	14.17	2.89	14.44	3.67	7.09	5.77	2.36	1.84	5.51	5.25	3.67	3.15	4.72	381
ND6	1.14	0.57	0.57	0.57	12.57	2.86	0.57	15.43	4.57	18.29	7.43	10.86	1.14	1.14	0.57	6.29	3.43	2.86	1.14	8.00	175
ND4L	1.09	1.09	1.09	4.35	10.87	4.35	3.26	15.22	3.26	18.48	11.96	3.26	0.00	1.09	1.09	9.78	0.00	5.43	0.00	4.43	92
ND4	3.15	2.02	0.90	2.02	9.66	6.07	1.35	9.66	0.36	17.30	10.56	4.94	2.47	1.35	1.12	8.99	1.80	3.15	2.70	7.19	445
ND5	3.29	0.87	2.25	1.91	9.36	4.16	0.69	10.75	3.81	16.64	9.88	5.72	1.91	1.04	0.87	11.44	2.60	4.51	1.56	6.76	577
ND3	1.72	1.72	2.59	3.45	11.21	1.72	1.72	19.83	6.03	12.96	5.17	6.03	3.45	0.86	1.72	10.34	3.45	1.72	2.59	1.72	116

Total	36.39	11.60	20.12	28.61	141.13	54.96	22.74	179.10	45.27	185.0	89.90	89.95	42.10	22.73	17.11	103.12	46.00	42.25	33.52	71.52	3698

**Table 6:** The codon usage of the skipper mt genome. *n*: frequency of codon used; RSCU: relative synonymous codon usage; *stop codon. Start codons and stop codons were excluded in total codon counts.

Codon (Aa)	*n* (RSCU)	Codon (Aa)	*n* (RSCU)	Codon (a)	*n* (RSCU)	Codon (Aa)	*n* (RSCU)
UUU (F)	351.0 (1.81)	UCU (S)	116.0 (2.71)	UAU (Y)	192.0 (1.90)	UGU (C)	34.0 (1.94)
UUC (F)	37.0 (0.19)	UCC (S)	16.0 (0.37)	UAC (Y)	10.0 (0.10)	UGC (C)	1.0 (0.06)
UUA (L)	458.0 (5.07)	UCA (S)	84.0 (1.96)	UAA (*)	0.0 (0.00)	UGA (W)	86.0 (1.87)
UUG (L)	13.0 (0.14)	UCG (S)	7.0 (0.16)	UAG (*)	0.0 (0.00)	UGG (W)	6.0 (0.13)
CUU (L)	45.0 (0.50)	CCU (P)	74.0 (2.45)	CAU (H)	55.0 (1.72)	CGU (R)	15.0 (1.20)
CUC (L)	6.0 (0.07)	CCC (P)	17.0 (0.56)	CAC (H)	9.0 (0.28)	CGC (R)	0.0 (0.00)
CUA (L)	19.0 (0.21)	CCA (P)	29.0 (0.96)	CAA (Q)	63.0 (1.97)	CGA (R)	31.0 (2.48)
CUG (L)	1.0 (0.01)	CCG (P)	1.0 (0.03)	CAG (Q)	1.0 (0.03)	CGG (R)	4.0 (0.32)
AUU (I)	431.0 (1.89)	ACU (T)	94.0 (2.54)	AAU (N)	209.0 (1.79)	AGU (S)	36.0 (0.84)
AUC (I)	26.0 (0.11)	ACC (T)	7.0 (0.19)	AAC (N)	25.0 (0.21)	AGC (S)	3.0 (0.07)
AUA (M)	245.0 (1.91)	ACA (T)	45.0 (1.22)	AAA (K)	106.0 (1.77)	AGA (S)	74.0 (1.73)
AUG (M)	12.0 (0.09)	ACG (T)	2.0 (0.05)	AAG (K)	14.0 (0.23)	AGG (S)	6.0 (0.14)
GUU (V)	61.0 (1.86)	GCU (A)	77.0 (2.44)	GAU (D)	58.0 (1.81)	GGU (G)	46.0 (1.00)
GUC (V)	1.0 (0.03)	GCC (A)	13.0 (0.41)	GAC (D)	6.0 (0.19)	GGC (G)	5.0 (0.11)
GUA (V)	61.0 (1.86)	GCA (A)	33.0 (1.05)	GAA (E)	71.0 (1.84)	GGA (G)	109.0 (2.37)
GUG (V)	8.0 (0.24)	GCG (A)	3.0 (0.10)	GAG (E)	6.0 (0.16)	GGG (G)	24.0 (0.52)
